# Assessment of Finite Rate Chemistry Large Eddy Simulation Combustion Models

**DOI:** 10.1007/s10494-017-9823-0

**Published:** 2017-06-13

**Authors:** E. Fedina, C. Fureby, G. Bulat, W. Meier

**Affiliations:** 1 Defence Security Systems Technology, The Swedish Defence Research Agency – FOI, SE 147 25 Tumba, Stockholm Sweden; 2 Siemens Industrial Turbomachinery Ltd., Ruston House, Waterside South, Lincoln, LN5 7FD England UK; 3 Institut für Verbrennungstechnik, Deutsches Zentrum für Luft- und Raumfahrt (DLR), Pfaffenwaldring 38, 70569 Stuttgart, Germany

**Keywords:** Finite rate chemistry model, Comparison, Gas turbine, Combustion, LES

## Abstract

Large Eddy Simulations (LES) of a swirl-stabilized natural gas-air flame in a laboratory gas turbine combustor is performed using six different LES combustion models to provide a head-to-head comparative study. More specifically, six finite rate chemistry models, including the thickened flame model, the partially stirred reactor model, the approximate deconvolution model and the stochastic fields model have been studied. The LES predictions are compared against experimental data including velocity, temperature and major species concentrations measured using Particle Image Velocimetry (PIV), OH Planar Laser-Induced Fluorescence (OH-PLIF), OH chemiluminescence imaging and one-dimensional laser Raman scattering. Based on previous results a skeletal methane-air reaction mechanism based on the well-known Smooke and Giovangigli mechanism was used in this work. Two computational grids of about 7 and 56 million cells, respectively, are used to quantify the influence of grid resolution. The overall flow and flame structures appear similar for all LES combustion models studied and agree well with experimental still and video images. Takeno flame index and chemical explosives mode analysis suggest that the flame is premixed and resides within the thin reaction zone. The LES results show good agreement with the experimental data for the axial velocity, temperature and major species, but differences due to the choice of LES combustion model are observed and discussed. Furthermore, the intrinsic flame structure and the flame dynamics are similarly predicted by all LES combustion models examined. Within this range of models, there is no strong case for deciding which model performs the best.

## Introduction and Background

During the last three decades the use of Large Eddy Simulation (LES) in turbulent combustion research and engineering has increased considerably as evident from statistics of open literature journal publications. Two decades ago LES was almost exclusively employed to study geometrically simple laboratory combustors to obtain more in-depth information about unsteady flow and flame features, whereas now, LES is routinely used to analyze full-scale combustors, e.g. [1–3]. This is due, in part, to the rapid and continuous increase in high-performance computing, [4], and the programming paradigms necessary to use these resources in an efficient manner, [5]. Concomitantly, the increasing understanding of turbulence, combustion chemistry, and how turbulence and combustion chemistry interact, have made it possible to advance the modeling to a sufficiently high level to support the development of such high-fidelity LES models. This is enabled by improved experimental facilities, and the development of non-intrusive diagnostics, [6, 7], as well as the increased use of Direct Numerical Simulations (DNS), [8, 9], which in turn is highly dependent on the high-performance computing development. For engineering applications, the industrial standard is Reynolds Averaged Navier–Stokes (RANS) models, e.g. [10], due to its fast turnaround time and relative success in providing combustor exit temperature profiles. The strong dependence on the turbulence and combustion models, [11], and the limited ability of RANS models to predict unsteady combustion, makes LES more attractive.

Combustion LES is based on the reactive Navier-Stokes Equations (NSE) and employs low-pass filtering, [12], to eliminate the small-scale flow physics not resolved on the computational grid. This implies that the large-scale flow physics, containing most of the kinetic energy, is resolved, whereas the effects of the unresolved small-scale flow physics must be handled by subgrid models. It is commonly proposed, [13], that at least 85% of the total kinetic energy should be resolved for the small scale (more universal) flow physics to be modelled by Kolmogorov turbulence, [14–16], and its effects on chemical reactions and interfaces, [17–19]. Properly used, LES allows for higher fidelity than RANS, at a lower computational cost compared to DNS. For practical engineering systems the use of LES typically requires large grids, and for wall-bounded flows either wall-resolved LES or wall-modeled LES can be used. In wall-resolved LES the grid is successively refined as the wall is approached in order to resolve all dynamically relevant flow scales, whereas in wall-modeled LES a wall-model is employed to embody the effects of the near-wall flow physics eliminated by the low-pass filtering, [20, 21]. In combustion LES both the subgrid stress and flux terms, [12], and the combustion source terms, or more precisely the low-pass filtered reaction rates, [22–25], through which the combustion chemistry enters the LES equations, requires closure modeling. The subgrid stress and flux terms can be modeled using extended versions of the subgrid models for incompressible or compressible LES, [12, 26], whereas the combustion source terms require a fundamentally different modeling approach due to the different nature of the physics to be modeled, [22–25].

As implied in several recent studies, e.g. [27, 28], the choice of reaction mechanism used to describe the combustion chemistry is very important for the performance of LES. The choice of the reaction mechanism is commonly a trade-off between computational capability and accuracy. *Detailed reaction mechanisms* include all intermediate reaction steps, and should be capable of accurately predicting a range of combustion properties including the laminar flame speed, flame temperature, ignition delay time, and extinction strain-rate. These mechanisms typically involve hundreds or thousands, of reactions, and hundreds of species, e.g. [29, 30], and are generally considered too comprehensive for LES, unless used in flamelet libraries, e.g. [22–25]. In order to keep the reaction mechanisms sufficiently small for affordable LES it has until recently been common to use *global reaction mechanisms*, consisting of only a few reactions and species, e.g. [31, 32], whereas most recently the use of *skeletal reaction mechanisms* (with about 20 to 100 reactions and tens of species) have gained increasing acceptance, [2, 27, 28, 33], demonstrating improved agreement with experimental data, and possibilities of comparing flame structure topology with experimental data.

Besides the subgrid flow modeling and the modeling of the combustion chemistry, the ability to resolve and/or model the turbulent reaction front is the other major challenge in combustion LES. This is manifested by the filtered reaction rates in the species transport equations, which are directly related to the underlying (detailed, skeletal or global) reaction mechanisms. Depending on the type of combustion (non-premixed or premixed), the relative turbulence intensity (characterized by the Reynolds number, Re = *v* *ℓ*_*I*_/*ν*, where *ℓ*_*I*_ is the integral length-scale) and the relative rate of reaction, (characterized by the Damköhler and Karlowitz numbers, Da = *τ*_*I*_/*τ*_*C*_ and Ka = *τ*_*K*_/*τ*_*C*_, respectively, in which *τ*_*I*_, *τ*_*K*_ and *τ*_*C*_ are the integral, Kolmogorov and chemical time scales, respectively) different types of LES models can be formulated, [22–25]. Two main classes of LES combustion models can be distinguished: *flamelet models* and *finite rate chemistry models*, both containing sub-classes and many different models. *Flamelet models* assume that the flame is thin compared to the length scales of the flow, and the flame behaves like an interface between fuel and oxidizer (in non-premixed combustion), e.g. [34, 35], or between reactants and products (in premixed combustion), e.g. [36, 37]. This results in that the species equations can be replaced by equations for the mixture fraction, *z*, [34], or the reaction progress variable, *c*, [37], or a kinematic *G*-field, [36], depending on on which type of flame is considered. Due to the scale separation it is then convenient to decouple the flow and chemistry, represented by one-dimensional laminar flame, combined in a flamelet library, which is modified by the turbulence in a separate step before being used in the *c* or *G* equations to bestow the laminar and turbulent flame speeds. *Finite rate chemistry models* assume nothing about the flow or flame but attempt to solve the species equations using models for the low-pass filtered reaction rates. Many different finite rate chemistry models are available including thickened flame models, [38], localized turbulent scales models, [39–41], approximate deconvolution models, [42], presumed probability density function models, [43], transported probability density function models, [27, 44], conditional moment closure models, [45], and linear eddy models, [46], all with their own advantages and disadvantages.

Regardless of many successful LES investigations there have, in general, been only a few head-to-head comparative studies of LES combustion models, e.g. [47, 48]. Studies like those are however needed to further advance the LES modeling technique, and to quantify the predictive capabilities and identify the limitations of LES combustion modeling. Here, we will endeavor to provide an independent assessment of a representative selection of finite rate chemistry LES models for a typical gas turbine burner mounted in a high-pressure experimental optical combustion chamber. The particular configuration selected has previously been experimentally examined in a series of papers, [49–51], resulting in a rather comprehensive experimental database including velocity, temperature and major species data. This set-up has also previously been successfully simulated with LES using different codes and different finite rate chemistry LES combustion models, e.g. [28, 44]. Here, the main objective will be to assess the predictive capability of finite rate chemistry LES using one of the abovementioned codes but a larger spectrum of finite rate chemistry LES models. A secondary objective is to enhance our understanding of swirl-stabilized gas turbines flames in general, and their combustion chemistry in particular. To model the combustion chemistry as accurately as possible, following [27, 28], and to support the second objective, a skeletal methane-air reaction mechanism is employed.

## Combustor, Experimental Set-Up and Measuring Techniques

**Fig. 1:**
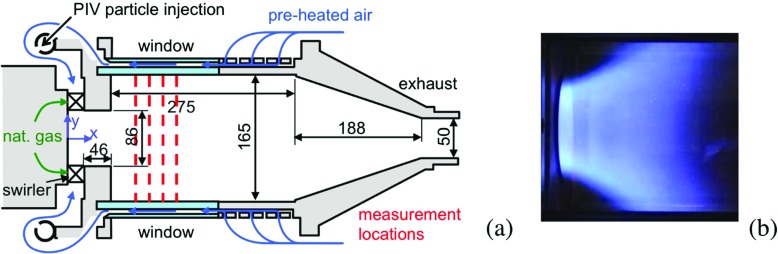
**a** Schematic of the SGT-100 burner and optical combustion chamber in high-pressure test rig at DLR Stuttgart based on Stopper et al. [49–51], and **b** a typical flame image

The SGT-100 Dry Low Emission (DLE) burner is the smallest combustor from the Siemens range with a power up to 1 MW. Six combustors are employed in the SGT-100 engine with a nominal power output of up to 5.7 MW for mechanical drive applications (twin-shaft) and a nominal power output of up to 5.4 MW for power generation (single shaft). In the present investigation, a full-scale single combustor was equipped with an optical combustion chamber and installed in a high-pressure test-rig at DLR Stuttgart. The flame reported on here was operated at 3 atm with an air temperature of 685 K, an air mass flow, including panel cooling, of 0.1749 kg/s, a fuel mass flow of 0.0062 kg/s, and a thermal power of 335 kW. It corresponds to Case A in [49–51]. The radial burner has multiple fuel injection holes that supply the fuel that mixes with the air whilst flowing through a pre-chamber of 46 mm length and D = 86 mm diameter according to Fig. 1. The mixture enters the combustor where an M-shaped flame is established in the annular region of shear between the internal and external flow zones.

**Fig. 2:**
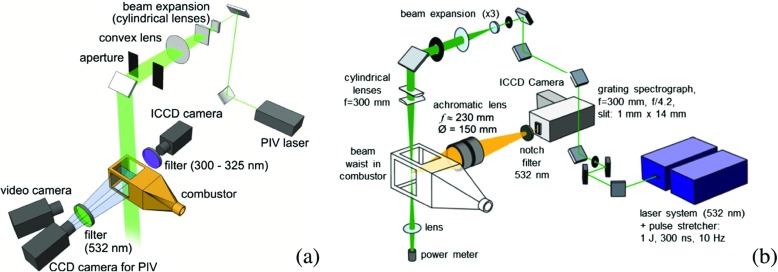
**a** Schematic of the experimental setup for the PIV measurement Also shown is the intensified CCD camera for the detection of the OH chemiluminescence and OH laser induced fluorescence and **b** experimental setup for the one-dimensional (1D) Raman measurements

The velocity was measured by Particle Image Velocimetry (PIV), Fig. 2a. TiO_2_ particles of 1 *μ* m diameter were seeded into the air flow and illuminated by a 1.0 mm thick light sheet generated by a frequency-doubled Nd:YAG laser (New Wave Solo PIV 120, *λ* = 532 nm). The two laser pulses for PIV were separated by 10 to 15 *μ* s. At a repetition rate of 5 Hz each pulse had an energy of 120 mJ and a duration of 5 ns. The particle distributions were detected by a CCD camera with 1376 × 1024 pixels (LaVision Imager Intense) equipped with a camera lens of 50 mm focal length. Image processing was carried out using DaVis 7.2 (LaVision GmbH). The spatial resolution of the measurement was 1.1 mm. Assuming a resolution of 0.1 pixels at the cross-correlation peak, the accuracy of the measured instantaneous velocities has a limit of 0.7 m/s. Because only one camera was employed for the PIV (not stereo-PIV) only in-plane velocities were measured. Therefore the perspective error was another source of uncertainty. Its value depends on the (unknown) out-of-plane velocity and the angle of view. Because the angle of view was small in the setup, the estimated error is smaller than the error caused by the pixel resolution. The typical total uncertainty is estimated to be on the order of ∼1 m/s.

The flame structures were measured by Planar Laser Induced Fluorescence (PLIF) of OH and OH chemiluminescence imaging, [50, 51], Fig. 2a. The OH-PLIF system consisted of a frequency-doubled dye laser (Lumonics HD-500) pumped by a frequency-doubled Nd:YAG laser (Quanta Ray DCR-2). The pulse length was 8 ns and the pulse energy 4.6 to 5.5 mJ. The laser wavelength was tuned to the Q_1_(8) line of the A-X (v = 1 ← v = 0) transition at *λ* ≈ 283.5 nm. In the combustor, the laser sheet thickness was about 0.4 mm. The fluorescence was detected by an intensified CCD camera (Princeton Instruments PI-Max, 512 × 512 pixels) using an achromatic UV lens (f = 100 mm, f/2) together with a band pass filter (*λ* = 300 − 325 nm). The imaged section in the flame’s axial direction was 59 mm, the spatial resolution 0.3 mm/pixel. The processing of the PLIF images included corrections for background, camera sensitivity and laser intensity profile. For the measurement of OH chemiluminescence, the same imaging system was utilized, and the instantaneous distributions were recorded with an exposure time of 40 *μ* s.

The joint PDFs of temperature, major species concentrations and mixture fraction were determined by one-dimensional laser Raman scattering. The Raman scattering system has been described previously, [50, 51], and only a brief summary is given here. The laser pulses from three double-pulse Nd:YAG lasers (Spectra Physics PIV 400), operated at *λ* = 532 nm, passed through a pulse stretcher that delivered a pulse train at its outlet of about 350 ns length, having a total pulse energy of 1 J. The beam was shaped by spherical and cylindrical lenses to form a beam waist of about 0.5 mm in the central part of the combustor, Fig. 2b. An 8 mm long section of the beam waist was relayed onto the entrance slit of a grating spectrograph (Acton-Research, SpectraPro 300i, f = 300 mm, f/4.2, grating: 490.4 lines per mm) by an achromatic lens system (Linos, f = 230 mm, aperture f/1.5). After spectral separation, the Raman signals from the major species (CH_4_, O_2_, N_2_, CO_2_, CO, H_2_O, H_2_) were detected by an intensified CCD camera (Princeton Instrument PI-Max, 1340 × 1300 pixels, with Gen III intensifier) with spectral resolution in one direction and spatial resolution in the other. The pixels were binned so that the measured line was divided into 28 observation volumes, each with 0.29 mm length. For the data reported here only the combined signal from the two central volumes are utilized. The species number densities were obtained from the Raman signal intensities using calibration measurements in laminar flames and flows. The temperature was deduced from the total number density via the ideal gas law. The measurement locations within the combustor were changed by translating the detectors and laser optics using a 2D translation stand. Radial profiles were measured at four axial locations, x/D = 1.21, 1.44, 1.66 and 2.00, where 500 single shot measurements were performed at each radial position y. The typical relative uncertainties of the mean values in the hot reaction products are 4% for *T* and 12% for $Y_{\text {CO}_{2}}$. In order to avoid vignetting the largest radial position accessible was y = 47.5 mm. During the measurements in the lean premixed flames it turned out that the species concentrations of H_2_ and CO were very small and below the detection limit of 0.5% by volume. Therefore, they were excluded from the data reduction and set to zero.

## LES Models and Numerical Methods

As mentioned in the introduction there are two main branches of combustion LES: flamelet LES, e.g. [34–37], and finite rate chemistry LES, e.g. [27, 38–46]. Regardless of branch, the governing equations are the low-pass filtered equations of mass, momentum and energy representing convection, diffusion and chemical reactions, [22–25]. The low-pass filtering is used to separate the resolved scale flow (denoted by $\widetilde {\text {tildes}}$ if density weighted filtering is used, and $\overline {\text {overbars}}$ if not) from the unresolved (subgrid) scale flow. The filtering is often implicit and implies that the physics on scales smaller than the filter width, Δ, must be represented by subgrid models and physics on scales larger than Δ are explicitly resolved. For a linear viscous reacting mixture with Fourier heat conduction and Fickian diffusion, the LES equations are,

1 $$ \left\{ \begin{array}{l} \partial_{t}(\bar{\rho})+\nabla\cdot(\bar{\rho}\tilde{\mathbf{v}}) = 0,\\ \partial_{t}(\bar{\rho}\tilde{Y}_{i})+\nabla\cdot(\bar{\rho}\tilde{\mathbf{v}}\tilde{Y}_{i})=\nabla\cdot(D_{i}\nabla \tilde{Y}_{i}-\mathbf{b}_{i})+\overline{\dot{w}_{i}},\\ \partial_{t}(\bar{\rho}\tilde{\mathbf{v}})+\nabla\cdot(\bar{\rho}\tilde{\mathbf{v}}\otimes\tilde{\mathbf{v}})=-\nabla\bar{p}+\nabla\cdot\left( 2\mu\tilde{\mathbf{D}}_{D}-\frac{2}{3}\mu(\nabla\cdot\tilde{\mathbf{v}})\mathbf{I}-\mathbf{B}\right),\\ \partial_{t}(\bar{\rho}\tilde{E})+\nabla\cdot(\bar{\rho}\tilde{\mathbf{v}}\tilde{E})=\nabla\cdot\left( -\bar{p}\tilde{\mathbf{v}}+2\mu\tilde{\mathbf{D}}\tilde{\mathbf{v}}-\frac{2}{3}\mu(\nabla\cdot\tilde{\mathbf{v}})\tilde{\mathbf{v}}+\kappa\nabla\tilde{T}-\mathbf{b}_{E}\right), \end{array} \right. $$

in which $\bar {\rho }$, $\tilde {\mathbf {v}}$, $\tilde {T}$ and $\tilde {Y}_{i}$ are the filtered density, velocity, temperature and species mass fractions, respectively, $\tilde {\mathbf {D}}=\frac {1}{2}\left (\nabla \tilde {\mathbf {v}} + \nabla \tilde {\mathbf {v}}^{\mathrm {T}}\right )$ the rate-of-strain tensor and $\tilde {\mathbf {D}}_{D}$ its deviatoric part. The filtered pressure is $\bar {p}\approx \bar {\rho }R\tilde {T}$, in which *R* is the (composition dependent) gas constant. The mixture is described by the viscosity *μ*, and the species and thermal diffusivities, *D*_*i*_ = *μ*/Sc_*i*_ and *κ* = *μ*/Pr, with Sc_*i*_ and Pr being the constant, species dependent, Schmidt and Prandtl numbers, respectively. The total energy $\tilde {E}=\tilde {e}+\frac {1}{2}\tilde {\mathbf {v}}^{2}+k$ is composed of the internal energy $\tilde {e} =\tilde {h}-\bar {p}/\bar {\rho }$, resolved kinetic energy $\frac {1}{2}\tilde {\mathbf {v}}^{2}$, and subgrid kinetic energy *k*, in which $\tilde {h} = {\sum }_{i}\left (\tilde {Y}_{i}\left (h_{i,f}^{\theta } + {\int }_{T_{0}}^{\tilde {T}} C_{p,i} \mathrm {d}T\right )\right )$ is the enthalpy, $h_{i,f}^{\theta }$ the formation enthalpies and *C*_*p*,*i*_ the specific heats. The flow physics associated with the small, unresolved, eddies is concealed in the subgrid stress and flux terms $\mathbf {B}=\bar {\rho }\left (\widetilde {\mathbf {v}\otimes \mathbf {v}}-\tilde {\mathbf {v}}\otimes \tilde {\mathbf {v}}\right )$, $\mathbf {b}_{i} =\bar {\rho }\left (\widetilde {\mathbf {v}Y_{i}} - \tilde {\mathbf {v}}\tilde {Y}_{i}\right )$ and $\mathbf {b}_{E} = \bar {\rho }\left (\widetilde {\mathbf {v}E} - \tilde {\mathbf {v}}\tilde {E}\right )$. The combustion chemistry enters (1_2_) through the filtered reaction rates $\overline {\dot {w}_{i}} = M_{i}P_{ij}\overline {\dot {w}_{j}}$, with *P*_*ij*_ being the stoichiometric coefficients, *M*_*i*_ the molar masses, and $\dot {w}_{j}=A_{j} T^{n_{j}}\exp \left (-T_{A,j}/T\right ){\prod }_{k=1}^{N}\left (\rho Y_{k}/M_{k}\right )^{b_{k}}$ the Arrhenius reaction rates. Here, *A*_*j*_ are the pre-exponential factors, *T*_*A*,*j*_ the activation temperatures, *n*_*j*_ the temperature exponent and *b*_*j*_ the reaction order for reaction *j*, [22–25].

The subgrid stress and flux terms need to be modeled in order to close the LES equations (1), and to represent the influence of the unresolved flow physics on the resolved flow physics. These terms can be divided into Leonard, cross and Reynolds stress and flux terms associated with interactions between the smallest resolved eddies, between the smallest resolved eddies and the largest unresolved eddies, and between the unresolved eddies, respectively, [52]. To embody this feature, the subgrid stress and flux terms are here modeled using mixed models, [53], so that $\mathbf {B}=\bar {\rho }\left (\widetilde {\tilde {\mathbf {v}}\otimes \tilde {\mathbf {v}}}-\tilde {\tilde {\mathbf {v}}}\otimes \tilde {\tilde {\mathbf {v}}}\right ) - 2\mu _{k}\tilde {\mathbf {D}}_{D}$, $\mathbf {b}_{i} =\bar {\rho }\left (\widetilde {\tilde {\mathbf {v}}\tilde {Y}_{i}} - \tilde {\tilde {\mathbf {v}}}\tilde {\tilde {Y}}_{i}\right ) - \frac {\mu _{k}}{\text {Sc}_{t}}\nabla \tilde {Y}_{i}$ and $\mathbf {b}_{E} = \bar {\rho }\left (\widetilde {\tilde {\mathbf {v}}\tilde {E}} - \tilde {\tilde {\mathbf {v}}}\tilde {\tilde {E}}\right ) - \frac {\mu _{k}}{\text {Pr}_{t}}\nabla \tilde {E}$ wherein $\mu _{k}=c_{k}\bar {\rho }{\Delta } k^{1/2}$, in which *k* is obtained from a modeled transport equation, [54], and Sc_*t*_ = Pr_*t*_ = 0.7.

Six different models for the low-pass filtered reaction rates, ${\overline {{{\dot {{w}}}_{{i}}}}}$, are here tested in order to portray the sensitivity of the finite rate chemistry LES model to the modeling of the low-pass filtered reaction rates, hence increasing our understanding of the modeling issues and aspects of the low-pass filtered reaction rates. The models tested can be summarized as:

- The Thickened Flame Model (TFM), [38], is based on the assumption that the flame can be thickened by decreasing the quasi-laminar reaction rates by a factor *F* = Δ/*δ*_*u*_, where Δ is the filter width and *δ*_*u*_ the laminar flame thickness, and increasing the diffusivity by *F* to preserve the laminar flame speed, *s*_*u*_. To allow for the increase in flame area due to turbulence, the quasi-laminar reaction rates and diffusivities, $\dot {w}_{i}(\bar {\rho }, \tilde {Y}_{i}, \tilde {T})$ and $D_{i}(\tilde {Y}_{i}, \tilde {T})$, respectively, are all pre-multiplied by the subgrid wrinkling factor, Ξ_Δ_, such that $\overline {\dot {w}_{i}}\sim {\Xi }_{\Delta }\dot {w}_{i}(\bar {\rho }, \tilde {Y}_{i}, \tilde {T})/F$ and $D_{i}\sim F{\Xi }_{\Delta } D_{i}(\tilde {Y}_{i}, \tilde {T})$, respectively. In this investigation, the dynamic TFM model of Legier et al. [55], is employed together with the semi-empirical power-law expression for the subgrid wrinkling factor Ξ_Δ_ proposed by Charlette et al. [56].
- The Eddy Dissipation Concept (EDC) model, [39], is a customized version for LES of the EDC model of Magnussen et al. [57–59]. The model is based on the assumption that combustion takes place in fine-structure regions characterized by intense chemical activity and vorticity, embedded in regions of lower levels of vorticity and chemical activity, [60]. The filtered reaction rates, $\overline {\dot {w}}_{i}$, are thus represented as weighted averages of the reaction rates in the fine-structures and surroundings so that $\overline {\dot {w}_{i}} = \gamma ^{\ast }\dot {w}_{i}^{\ast }+(1-\gamma ^{\ast })\dot {w}_{i}^{0}$, in which *γ* ^∗^ is the reacting volume fraction and $\dot {w}_{i}^{\ast } = \dot {w}_{i}^{\ast }(\bar {\rho }, Y_{i}^{\ast }, T^{\ast })$ and $\dot {w}_{i}^{0} = \dot {w}_{i}^{0}(\bar {\rho }, {Y_{i}^{0}}, T^{0})$ are the reaction rates in the fine-structures and surroundings, respectively. Since the filtered species mass fractions and temperature are $\tilde {Y}_{i} = \gamma ^{\ast } Y_{i}^{\ast } + (1 - \gamma ^{\ast }){Y_{i}^{0}}$ and $\tilde {T} = \gamma ^{\ast } T^{\ast } + (1 - \gamma ^{\ast })T^{0}$, local subgrid balance equations of mass and energy of the form $\bar {\rho }(Y_{i}^{\ast } - \tilde {Y}_{i}) = (1 - \gamma ^{\ast })\tau ^{\ast }\dot {w}_{i}(\bar {\rho }, Y_{i}^{\ast }, T^{\ast })$ and $\bar {\rho }{\sum }_{i=1}^{N} \left (Y_{i}^{\ast } h_{i}^{\ast }(T^{\ast }) - \tilde {Y}_{i}\tilde {h}_{i}(\tilde {T})\right ) = (1 - \gamma ^{\ast })\tau ^{\ast }{\sum }_{i=1}^{N} h_{i,f}^{\theta }\dot {w}_{i}(\bar {\rho }, Y_{i}^{\ast }, T^{\ast })$ can be solved for $Y_{i}^{\ast }$, ${Y_{i}^{0}}$, *T* ^∗^ and *T* ^0^ provided estimates of the reacting volume fraction *γ* ^∗^ and the subgrid time scale *τ* ^∗^. In general $\dot {w}_{i}^{\ast }\gg \dot {w}_{i}^{0}$ such that $\dot {w}_{i}^{0}$ may be neglected. Here, *γ* ^∗^ and *τ* ^∗^ are both estimated using the cascade process, [57, 58], resulting in that $\gamma ^{\ast } = 1.02(\nu /{\Delta } {v}^{\prime })^{3/4}$ and $\tau ^{\ast } = 1.24({\Delta }\nu /{v^{\prime }}^{3})^{1/2}$, in which *ν* is the molecular viscosity and $v^{\prime }=\sqrt {2k/3}$ the subgrid velocity fluctuations.
- The Fractal Model (FM), [40], is based on the same assumptions as the EDC model, [39], but the reacting volume fraction, $\gamma ^{\ast }$, is here estimated using an assumed fractal-like behavior of the fine structures. This results in that $\gamma ^{\ast }=\gamma _{N}({\Delta }/\ell _{K})^{D_{3}-2}$, in which *γ*_*N*_ = *N*_*K*_/*N*_*T*_ is the ratio of the number *N*_*K*_ of Kolmogorov (*ℓ*_*K*_) scales to the total number of scales, *N*_*T*_, generated locally and *D*_3_ is the local fractal dimension. In order to estimate *γ*_*N*_, a model must be adopted to control the fractal generation process, and in [40] an analytical fit of the form $\gamma _{N}\approx 1-\left (\left (0.36({\Delta }/\ell _{K} - 1)\right )/\left (1 + 0.0469({\Delta }/\ell _{K} - 1)^{2.7}\right )\right )$ is proposed. The fractal dimension is estimated using a box-counting method, [61], so that $D_{3} = 3-\left (\log (\pi )/\log ({\Delta }/\ell _{K})\right )$. Combining these expressions we find that *γ* ^∗^ is a function of the ratio of Δ to *ℓ*_*K*_ which has an asymptotic value of *γ* ^∗^ = 1/*π* ≈ 0.318 for high cell Re-numbers, Re_Δ_ = |**v**|Δ/*ν*.
- The Partially Stirred Reactor (PaSR) model, [41], is also based on the same assumptions as the EDC model, [39], but the reacting volume fraction, *γ* ^∗^, is here estimated from theoretical estimates, [5, 6], and DNS data, e.g. [60]. The modeling of the reacting volume fraction is based on either a time-series analysis or a geometrical analysis in which the reacting fine structures are lumped together such that *γ* ^∗^≈ *τ*_*C*_/(*τ* ^∗^ + *τ*_*C*_). The chemical time-scale represents the overall combustion reaction, and is thus represented by *τ*_*C*_ ≈ *δ*_*u*_/*s*_*u*_. The modeling of *τ* ^∗^ is based on the observation that the fine structure area-to-volume ratio is given by the dissipative length scale, $\ell _{D} = \left (\nu /(v^{\prime }/{\Delta })\right )^{1/2}$, and that the velocity influencing these is the Kolmogorov velocity, *v*_*K*_, such that *τ* ^∗^ = *ℓ*_*D*_/*v*_*K*_. Evaluating this expression results in that $\tau ^{\ast } = \sqrt {\tau _{K}\tau _{\Delta }}$, in which *τ*_*K*_ is the Kolmogorov time-scale, and $\tau _{\Delta }={\Delta }/{v}^{\prime }$ the shear time-scale, representative of dissipation and small-scale mixing, [62]. By combining the aforementioned expressions for *τ* ^∗^ and *τ*_*C*_ it is found that $\gamma ^{\ast }\approx \beta (v^{\prime }/s_{u})^{5/4}/\left (({\Delta }/\delta _{u})^{3/4} + \beta (v^{\prime }/s_{u})^{5/4}\right )$, where *β* ≈ 1.17.
- The Approximate Deconvolution Model (ADM) was developed for non-reactive flows by Stoltz and Adams [46], and was expanded to reactive flows by Mathew, [42]. Given a filter, *G*, an approximate inverse filter operator can be constructed so that $G^{-1}\approx Q_{N}={\sum }_{\nu =0}^{N}(I-G)^{\nu }$, in which *I* is the identity operator. For an arbitrary field, *f*, this implies that $f^{\ast }=G^{-1}\ast \tilde {f}\approx Q_{N}\ast \tilde {f}$ is an approximation (of order *N*) to *f*. This results in an opportunity to provide a direct closure to the low-pass filtered reaction rates according to $\overline {\dot {w}_{j}}\approx \overline {A_{j}(T^{\ast })^{n_{j}}\exp \left (-T_{A,j}/T^{\ast }\right ){\prod }_{k=1}^{N}\left (\rho ^{\ast } Y_{k}^{\ast }/M_{k}\right )^{b_{k}}}$ and all other subgrid terms, such as for example $\mathbf {B} = \bar {\rho }(\widetilde {\mathbf {v}^{\ast }\otimes \mathbf {v}^{\ast }} - \tilde {\mathbf {v}}\otimes \tilde {\mathbf {v}})$. In this study, however, the ADM approach in only applied to the low-pass filtered reaction rates in order to facilitate a direct comparison of the effects of different reaction rate models. A similar approach based on Taylor series has recently been proposed, [63].
- The transported Probability Density Function (PDF) class of models, [25, 27, 44], consists of several sub-classes of models of which the most known are the Lagrangian PDF model, [27, 45], and the Stochastic Fields (SF) model, [44, 64]. An equation describing the evolution of the PDF, *P*, can be derived by standard methods, e.g. [65]. This equation includes unknown terms, representing subgrid transport and subgrid mixing. Here, these are represented, respectively, by a gradient model and by the linear mean square estimation model, [66]. With these models incorporated the PDF equation becomes,
  2 $$\begin{array}{@{}rcl@{}} \partial_{t}(\bar{\rho}P)+\nabla\cdot(\bar{\rho}\tilde{\mathbf{v}}P) &=& \nabla\cdot\left( \left( \frac{\mu}{\sigma} + \frac{\mu_{k}}{\sigma_{k}}\right)\nabla P\right) + \frac{C_{d}}{\tau_{k}}\sum\limits_{\alpha=1}^{N}\partial_{\psi_{\alpha}}\left( \bar{\rho}\left( \psi_{\alpha} - \tilde{\phi}_{\alpha}\right)P\right)\\ && - \sum\limits_{\alpha=1}^{N_{s}}\partial_{\psi_{\alpha}}\left( \bar{\rho}\dot{w}_{\alpha}(\psi)P\right), \end{array} $$
  where *ϕ* is the random variable and *ψ* the sample space variable corresponding to *ϕ*, and *σ* = *σ*_*k*_ = 0.7, and *C*_*d*_ = 2. The micro-mixing time is obtained from $\tau _{k} = \bar {\rho }{\Delta }^{2}/(\mu +\mu _{k})$ and the number of scalar quantities, *N*, is equal to the number of species considered plus one. Here, the SF approach is used to solve (2) whereby *P* is represented by an ensemble of *N*_*s*_ stochastic fields with each field encompassing the *N* scalars, $\xi _{\alpha }^{n}$ for 1 ≤ *n* ≤ *N*_*s*_ and 1 ≤ *α* ≤ *N*. Following [27] the Ito formulation of the stochastic integral is adopted so that,
  3 $$\begin{array}{@{}rcl@{}} \bar{\rho}\mathrm{d}\xi_{\alpha}^{n}+\bar{\rho}\tilde{\mathbf{v}}\cdot\nabla\xi_{\alpha}^{n}\mathrm{d}t &= & \nabla\cdot({\Gamma}\nabla\xi_{\alpha}^{n})\mathrm{d}t + \bar{\rho}\sqrt{\frac{2{\Gamma}}{\bar{\rho}}}\nabla\xi_{\alpha}^{n}\cdot\mathrm{d}\mathbf{W}^{n} -\frac{1}{2}\bar{\rho}\frac{C_{d}}{\tau_{k}}(\xi_{\alpha}^{n} - \tilde{\phi}_{\alpha})\mathrm{d}t\\ &&+ \bar{\rho}\dot{w}_{\alpha}^{n}(\xi_{\alpha}^{n})\mathrm{d}t, \end{array} $$
  where Γ is the total diffusion coefficient and $\mathrm {d}\mathbf {W}_{i}^{n}$ the increments of a Wiener process, different for each field but independent of the spatial location. The stochastic fields given by Eq. 3 form an equivalent stochastic system to Eq. 2, smooth over the scale of the filter width. Again following [44], eight realizations are solved for each field.

The combustion LES models studied here are implemented in OpenFOAM, [67], and the equations are solved using a high-order monotonicity preserving convective reconstruction algorithm, central differencing and Crank-Nicholson time-integration, [68]. The combustion chemistry is integrated in time using a Strang-type, Rosenbrock, operator-splitting scheme, [69]. A fully compressible Pressure-based Implicit Splitting of Operators (PISO), [70], method is used for the pressure-velocity-density coupling. Stability is enforced using compact stencils, and by enforcing conservation of kinetic energy with a Courant number < 0.5.

The computational model of the SGT-100 combustor is constructed using block-structured hexahedral grid. The hexahedral grids were clustered around the swirler, flame, and at the combustor walls. Dirichlet conditions are used for all variables except for the pressure, *p*, at the inlet. At the outlet, all variables, except *p*, are extrapolated, whereas *p* is subject to wave-transmissive boundary conditions, [71]. At the walls, a no-slip wall-model, [72], is used together with zero Neumann conditions for all other variables. The fuel is German natural gas, which here is modeled as 98.97% CH_4_, 0.81% N_2_ and 0.21% CO_2_. The LES are initialized using a steady-state RANS result with a superimposed combustion region and are continued until the statistical moments has settled. The baseline grid contains 7 million cells but a finer grid with 56 million cells, has been used to study grid resolution effects. The results have been tested against the LES Index of Quality, [73], from which it was found that 87% and 93%, respectively, of the kinetic energy was resolved for the two grids, rendering both appropriate for LES.

## Chemical Kinetics

**Fig. 3:**
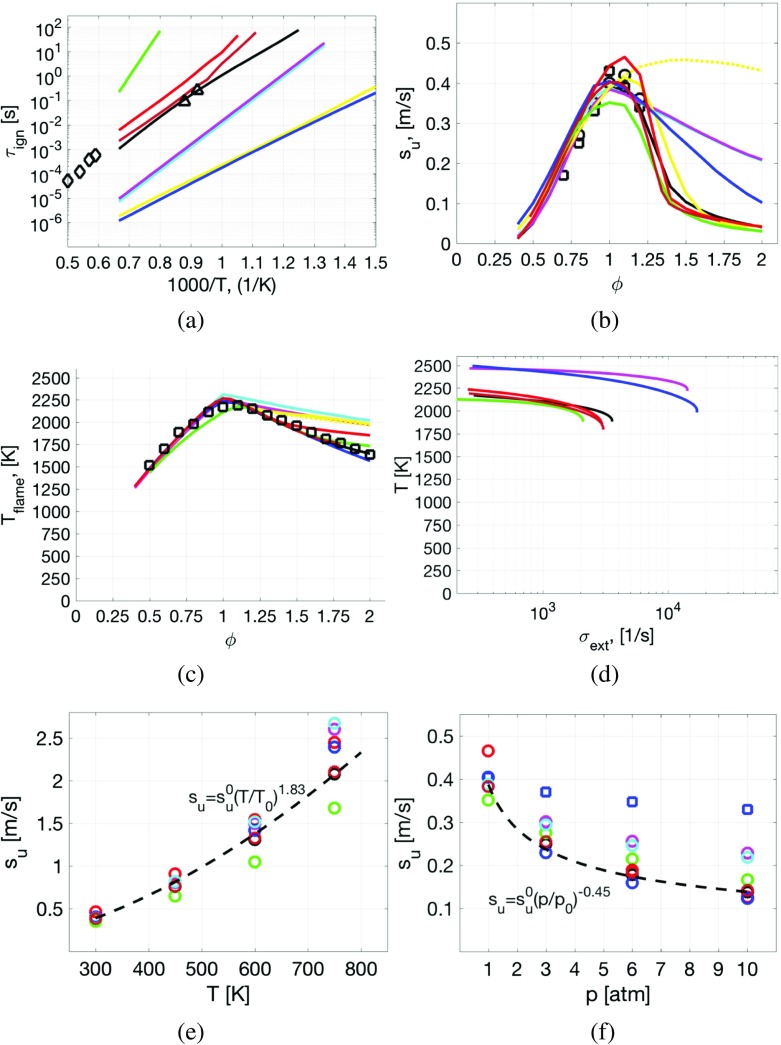
Comparison of **a** ignition delay times, *τ*_*ign*_, **b** laminar flame speeds, *s*_*u*_, and **c** flame temperature, *T*_*flame*_, and **d** extinction strain rates, *σ*_*ext*_, at *p* = 1 atm and 300 K, whereas **e** shows the variation of *s*_*u*_ with temperature, *T*, and **f** the variation of *s*_*u*_ with pressure, *p*. Legend: () WD1, [31], () WD2, [31], () JL4, [32], () 2sCM2, [74], () 2sCM2v, [74], () SG35, [75], () SR39, [76], () Z42, [77], (—) GRI-3.0, [29]; (○, □) experimental data from [78], (◇) experimental data from [79] and (△) experimental data from [80]. In **e** and **f**, () refers to the riginal JL4 mechanism, [32], whereas () refers to the modified JL4 mechanism, [28] and the *dashed line* corresponds to the curve fits based on the GRI–30 mechanism provided (for better representation of colour the reader is referred to the web-version of the paper)

In this study, six reaction mechanisms of increasing complexity are used to examine the sensitivity of the reaction mechanism on the laminar flame properties. These mechanisms are in order of generality and number of reactions and species: the 1- and 2-step global reaction mechanisms of Westbrook and Dryer [31], WD1 and WD2 respectively; the 2-step global reaction mechanism of Selle et al. [74], without, 2sCM2, and with, 2sCM2v, pre-exponential factor adjustment; the 4-step global reaction mechanism of Jones and Lindstedt [32], JL4; the 35-step skeletal reaction mechanism of Smooke and Giovangigli [75], SG35; the 39-step skeletal reaction mechanism of Sher and Refael [76], SR39; the 42-step skeletal reaction mechanism proposed by Zettervall et al. [77], Z42; and the comprehensive GRI-3.0 reaction mechanism, [29]. The JL4 reaction mechanism is, following the study of Bulat et al. [28], slightly modified to better handle the influence of pressure, *p*, on the laminar flame speed, *s*_*u*_, by allowing the pre-exponential factors, *A*_*k*_, to depend on pressure, *p*, so that $A_{k}=A_{k}^{p_{0}}(p/p_{0})^{-0.865}$, in which *p*_0_ = 1.013 atm. Figure 3a to d compare these mechanisms for laminar premixed flames at 1 atm and 300 K, and Fig. 3e and f show the dependence of *s*_*u*_ for various inflow temperatures, *T*, and pressures, *p*.

From Fig. 3a, comparing ignition delay times, *τ*_*ign*_, we find a large spread between reaction mechanism predictions, with JL4 and SR39 showing the largest deviations from the GRI-3.0 reaction mechanism, and SG35 and Z42 the best overall agreement. The laminar flame speed, *s*_*u*_, in Fig. 3b, shows that the experimental data of Vagelopoulos and Egolfopoulos [78], agrees well with the reference GRI-3.0 reaction mechanism predictions, and that the global WD1, WD2 and JL4 reaction mechanisms all fail to predict the fall-off of *s*_*u*_ for rich flames. This is due to the absence of intermediate species, including several C-based species, also resulting in overprediction of the flame temperature. The three skeletal reaction mechanisms SG35, SR39 and Z42 perform well over the range of equivalence ratios, 0.4 < *ϕ* < 2.0 considered, with Z42 showing the best overall agreement with the experimental data and the reference GRI-3.0 reaction mechanism predictions. The flame temperature is presented in Fig. 3c, and generally reveals acceptable agreement between global, skeletal and detailed mechanisms and experimental data on the lean side, whereas significant differences occur on the rich side. This is consistent with the *s*_*u*_ predictions, and is caused by the absence of intermediate species, in particular the C-based species. The extinction strain rates, *σ*_*ext*_, are presented in Fig. 3d, and reveal that all three skeletal reaction mechanisms are in agreement with the GRI-3.0 reaction mechanism, whereas the global reaction mechanisms all overpredict *σ*_*ext*_, resulting in incorrect turbulent flame predictions. The variation of *s*_*u*_ with pressure, *p*, and temperature, *T*, are shown in Fig. 3e and f, respectively, and shows that only the skeletal reaction mechanisms SG35, Z42, and to some extent also SR39, reproduce the variation of *s*_*u*_ with *p* and *T*, being crucial for successful gas turbine combustion modeling. Based on Fig. 3, Z42 presents the best agreement with the reference GRI-3.0 mechanism and the experimental data, followed by SG35 and SR39. In the subsequent simulations,the SG35 reaction mechanism is used to maintain consistency with Bulat et al. [28].

## Results and Discussion

**Fig. 4:**
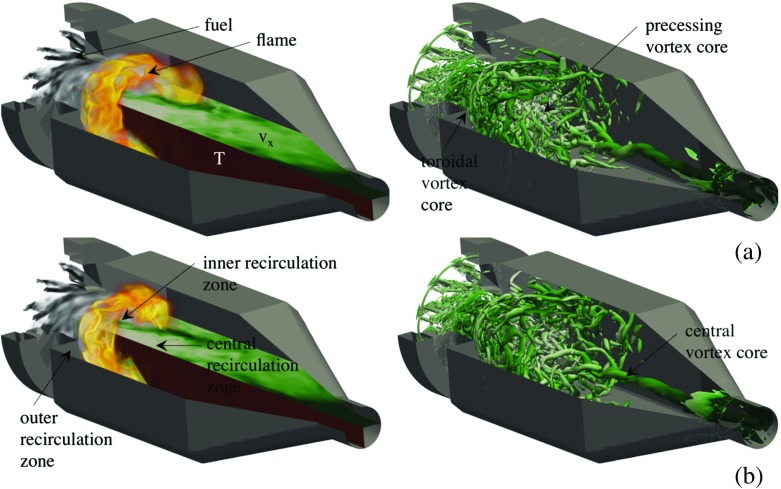
Instantaneous volumetric rendering of CH_4_ (*gray*) and the heat release, *Q*, (*orange*) together with axial velocity (*green*) and temperature (*red*) (*left panels*) and the second invariant of the velocity gradient tensor, *λ*_2_, colored by the axial velocity (*right panels*) for **a** LES-PaSR with the SG35 reaction mechanism and **b** LES-SF with the SG35 reaction mechanism. The color shadings for *Q* ranges from *opaque red* to *semi-transparent white* and for $\protect Y_{\text {CH}_{4}}$ from *opaque black* to *semi-transparent gray* using a linear mapping from 5 to 95% of the peak value in the volume (for better representation of colour, the reader is referred to the web-version of the paper)

Figure 4a and b show instantaneous volumetric renderings of the CH$_{4}$ mass fraction, $Y_{\text {CH}_{4}}$, (in gray) and the heat-release, *Q*, (in orange), representative of the luminous flame, contours of the axial velocity, *v*_*x*_, (in green) and the temperature, *T*, (in red) on two orthogonal half-planes, and an iso-surface of the second invariant of the velocity gradient tensor, *λ*_2_, colored by *v*_*x*_, from the LES-PaSR and LES-SF model predictions, respectively. The results from these two LES model predictions represent well the spectrum of predictions from all LES models examined, with variations mainly related to the size, shape, dynamics and flame intensity, and the size and shape of the inner and outer recirculation zones as well as the high-speed swirling velocity funnel exiting from the burner. Within the SGT-100 burner, the fuel discharges into the twelve radial swirlers, Fig. 1, where it immediately starts to mix with the radially supplied air before entering the pre-chamber at a fixed angle. This results in a strongly swirling flow, with most of the fuel located in a region flanking the pre-chamber wall, where it continues to mix with the air until the now well-mixed, wrinkled and furrowed, M-shaped fuel-air mixture discharges into the combustor, having a protracted inner V structure extending well into the pre-chamber. A flame develops around the M-shaped fuel-air cloud when this touches the hot combustion products in the inner and outer re-circulation zones, and otherwise along the boarder of the fuel-air cloud.

**Fig. 5:**
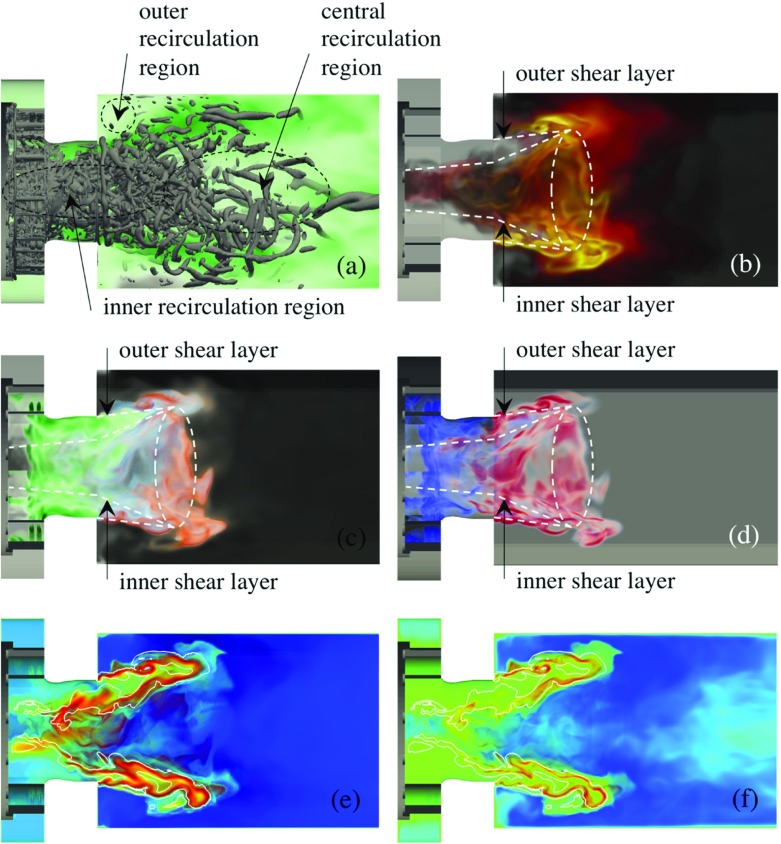
Results from LES-PaSR using the SG35 mechanism in terms of **a** volumetric renderings of the axial velocity, *v*_*x*_, and iso-surfaces of the second invariant of the velocity gradient tensor, *λ*_2_, **b** volumetric renderings of the heat-release, *Q*, **c** volumetric renderings of CH_4_ (*green*), CH_2_O (*blue*), HO_2_ (*purple*), HCO (*red*), CO (*orange*) and CO_2_ (*gray*), **d** volumetric renderings of the Takeno Flame Index, TFI, and volumetric renderings of the chemical explosive mode **e** eigenvalue, *λ*_*e*_, and **f** Damköhler number, Da_*e*_. The color shading for *v*_*x*_ ranges from *white* to *green*, for *Q* the color shadings ranges from *opaque black* via *yellow* and *red* to *semi-transparent white*, for the species, the color shadings ranges from the opaque color of the specie to semi-transparent white, for TFI the color shading ranges from *opaque blue* via *semi-transparent white* to *opaque red*, and for the two CEMA variables, $\protect \lambda _{e}$, and Da_*e*_, the color shading ranges from *semi-transparent blue* via *green* and *yellow* to *opaque red*. The white line in Fig. 5e and f corresponds to *λ*_*e*_ = 0 (and therefore also Da_*e*_ = 0). For all variables, a linear mapping from 5 to 95% of the peak value in the volume is used (for better representation of colour the reader is referred to the web-version of the paper)

The luminous flame, or rather the heat-release, $Q={\sum }_{i=1}^{N} h_{i,f}^{\theta }\overline {\dot {w}_{i}}$, consequently takes the shape of a strongly wrinkled M-shaped structure that wraps around the premixed fuel-air cloud as it turns around the combustor axis due to the imposed swirl. The heat-release results from multiple thin layers of inter-penetrating chemical reaction structures and layers of intermediate and radical species defined by the reaction mechanism (see also Fig. 5). The flame is located inside the combustion chamber, without any apparent wall attachment, and consists of two parts: an inner, strongly wrinkled, V-shaped swirling flame, found in the inner annular shear-layer of the discharging fuel-air mixture, reaching far into the pre-chamber, occasionally attaching to the rear burner surface, and an outer swirling flame, taking the shape of a truncated wrenched cone located in the outer annular shear-layer of the discharging fuel-air mixture. These two flame elements connect to each other at the swirling annular flame tip, located between 0.5D and 0.8D downstream of the burner exit plane depending on LES combustion model, but also on the flame dynamics. The fluid strain in the inner and outer annular shear-layers is observed to be highly intermittent, and sometimes in excess of the extinction strain rate of the SG35, SR39, Z42 and GRI-3.0 reaction mechanisms, but not in excess of the extinction strain rate of the global WD1, WD2 and JL4 mechanisms.

From the velocity distributions in the left panels of Fig. 4a and b, respectively, and the vorticity distributions in the right panels of Fig. 4a and b, respectively, we find high velocity magnitudes in the annular fuel-rich shear-layer discharging from the burner as well as in the contraction part and in the outlet section. The flow in the combustor develops into three recirculation regions (i) an outer recirculation region formed between the discharging fuel-air mixture and the combustor dump plane and the combustor wall; (ii) an inner recirculation region resulting from the axisymmetric vortex breakdown, [81], and (iii) a weak central recirculation region dominated by the exit confinement. In particular, is the inner reversed flow zone attached to the rear surface of the burner (see also Fig. 5), establishing a firm aerodynamic base for flame-stabilization. The vorticity distribution is very complicated, in particular in the burner and pre-chamber but also around the flame where annular vortex structures recently shed off the sharp edges of the burner can be seen traversing downstream whilst breaking up. Fragments of the toroidal vortex, around which the outer recirculation region is formed, can also be observed. In addition to these vortical structures, the two dominating vortical structures are the precessing vortex core, consisting of a single helical structure (partly hidden by the shear layer vortices), the breakdown of which results in the inner recirculation region, and the central vortex core, [82], formed at the rear end of the central recirculation region and extending all the way to the outlet.

Figure 5 provides more detailed information on the flow, flame, species, flame characteristics and turbulence chemistry interactions in the upstream part of the combustor. For this purpose we use the predictions from the LES-PaSR model, being a reasonable representative of the models tested here. These panels show side views of the combustor with volumetric renderings of (a) *v*_*x*_ and *λ*_2_, (b) *Q*, (c) methane (CH_4_), formaldehyde (CH_2_O), formyl (HCO), hydroperoxyl (HO_2_), carbon monoxide (CO) and carbon dioxide (CO_2_) mass fractions, *Y*_*i*_, (d) the Takeno flame index, [83], and the chemical explosive mode, [84, 85], (e) eigenvalue, *λ*_*e*_, and (f) Damköhler number, Da_*e*_. From Fig. 5a, the swirling high-speed velocity cone, discharging from the burner, can be seen together with the inner-, outer and central recirculation regions. The start of the central vortex core, adhering to the end of the central recirculation region, can also be seen together with annular Kelvin-Helmholz vortices shed off the burner exit, and multiple vortices from the fuel injectors and inner shear layers. The precessing vortex core is mainly hidden by the vortical structures from the fuel injectors and inner shear layers. The heat-release, *Q*, in Fig. 5b, reveals a complex structure with ridges of high *Q* within the inner and outer swirling annular shear layers, and along the swirling annular flame tip. The ridges correspond to regions where matching reactant-pairs (e.g. CH_4_ and H, and CH_4_ and OH) are irregularly co-located, facilitating exothermic reactions. The flow and species transport properties govern the mixing that facilitates the co-location of the reactant-pairs, and thus indirectly the exothermicity. Note that both the inner and outer annual shear-layers show instances of local quenching due to excessive strain-rates, resulting in spatial variations of *Y*_*i*_, which in turn results in variations of $\overline {\dot {w}_{i}}$ and *Q*. Figure 5c presents the corresponding CH_4_, CH_2_O, HO_2_, HCO, CO and CO_2_ mass-fraction distributions, which also reveal comparable ridges. It is evident that some (intermediate) species (e.g. CH_2_O) that participate in the reactions exist in the high strain-rate regions (e.g. the inner and outer shear layers) whereas others (e.g. HCO, HO_2_ and CO) do not, implying that turbulence, and particularly strain, modifies the reaction paths. The species typically exist in thin furrowed layers with CH_4_ forming the core together with O_2_, and CO and CO_2_ outermost. Also, CO_2_ forms downstream of CO as a direct consequence of CO+OH$\leftrightarrow \textit {CO}_{2}$+H, following the staged reaction process.

The Takeno Flame Index (TFI), [83], shown in Fig. 5d, is a metric for the alignment between the gradient of the CH_4_ and O_2_ mass-fractions used to describe the mixing mode between the CH_4_ and O_2_ such that $G=\nabla Y_{\text {CH}_{4}}\cdot \nabla Y_{\mathrm {O}_{2}}$. Domingo et al. [86], in their investigation of partially premixed gaseous and spray flames, normalized *G* such that *ξ* = *G*/|*G*| so *ξ* > 0 in premixed reaction zones and *ξ* < 0 in non-premixed reaction zones. This means that in premixed reaction zones (with *ξ* > 0) CH_4_ and O_2_ are consumed in the same physical direction, whereas in non-premixed reaction zones (with *ξ* < 0) CH_4_ and O_2_ are consumed in opposite directions. The topological nature of the annular M-shaped flame is evident from Fig. 5d, with non-premixed regions most frequently occurring within the inner shear layer of the pre-chamber, and premixed regions most frequently occurring in the part of the flame that resides in the combustor.

The Chemical Explosives Mode Analysis (CEMA), [84], is an alternative method based on eigenvalue analysis of the chemical source term Jacobian to identify critical flame topologies. In CEMA the species and energy equations are reformulated as evolution equations for the associated eigenmodes. Eigendecomposition of the chemical source term Jacobian renders the chemical modes, **f**_*i*_, and their eigenvalues, *λ*_*i*_. Following [84], negative eigenvalues correspond to chemical modes evolving towards equilibrium, whereas positive eigenvalues define explosive modes with a characteristic timescale $\lambda _{i}^{-1}$. Based on [85], the least-negative eigenvalue, aside from the zero eigenvalue associated with the conservation modes, is hereafter denoted *λ*_*e*_, corresponding to the fastest explosive mode. By comparing *λ*_*i*_ to the subgrid time-scale, *τ*_*k*_, an effective Da number can be defined as Da_*i*_ = *τ*_*k*_ *λ*_*i*_, providing a relation between the combustion chemistry and the turbulence. To clarify the visualization of the CEMA variables, we here consider signed log-scalings, [84, 85], and show renderings of $\lambda _{e}^{\ast } = \text {sgn}(\lambda _{e})\log _{10}\left (\max (\lambda _{e}, 1.0)\right )$ in Fig. 5e, and $\mathrm {Da_{e}^{\ast }}=\text {sgn}(\mathrm {Da_{e}})\log _{10}\left (\max (|\mathrm {Da_{e}}|, 1.0)\right )$ in Fig. 5f. From Fig. 5e a strong explosive mode ($\lambda _{e}^{\ast } > 0$) is observed along the inner- and outer shear layers, indicating the M-shaped flame. Outside of this, non-explosive ($\lambda _{e}^{\ast } < 0$) modes dominate both in the cold fuel-air mixing regime in the pre-chamber and in the hot combustion product regime in the combustor. The $\mathrm {Da_{e}^{\ast }}$ distribution in Fig. 5f resembles that of $\lambda _{e}^{\ast }$ in Fig. 5e, and clearly reveals an M-shaped flame located in the inner- and outer shear layers with three distinct regimes: (i) $\mathrm {Da_{e}^{\ast }} > 1$, with *τ*_*e*_(= 1/*λ*_*e*_) ≪ *τ*_*k*_, dominates in the premixed flame, (ii) $\mathrm {Da_{e}^{\ast }}\approx 0$, with *τ*_*e*_(= 1/*λ*_*e*_) ≈ *τ*_*k*_, dominates in the mixing and discharging fuel-air mixture surrounding the flame, and (iii) $\mathrm {Da_{e}^{\ast }} < 1$, with *τ*_*e*_(= 1/*λ*_*e*_) ≫ *τ*_*k*_, dominates in the hot combustion product regime. The solid white line in Fig. 5e and f, defined by *λ*_*e*_ = 0, denotes the sharp edge between explosive and non-explosive mixtures.

**Fig. 6:**
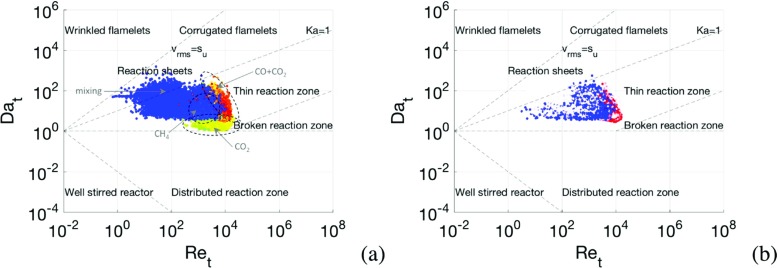
Turbulent combustion regime diagrams based on Libby and Williams [87], colored by **a** the heat release, *Q*, and **b** by theTakeno Flame Index (TFI). Colour range from **a** *blue* through *yellow* to *red* and **b** from *blue* through *white* to *red*

To assess the performance of the LES combustion models, it is useful to have an indication of the combustion regimes to be expected. Following Libby and Williams [87], we consider a combustion diagram based on the turbulent Da and Re numbers, D*a*_*t*_ = *τ*_*t*_/*τ*_*c*_ and $\mathrm {Re_{t}}_=v^{\prime }\ell _{t}/\nu $, respectively, in which $\tau _{t}=\ell _{t}/v^{\prime }$ is the turbulent time-scale, $\tau _{c}=\nu /{s_{u}^{2}}$ the chemical time-scale, *ℓ*_*t*_ the turbulent length scale and *v*′ the rms-velocity fluctuations. For Da_*t*_ > 1, *τ*_*t*_ > *τ*_*c*_, and *reaction sheets* form, whereas for Da_*t*_ < 1 the turbulent scales rapidly mix the species, which leads to *distributed reaction zones*. If both scales are similar, Da_*t*_ ≈ 1, strong turbulence-chemistry interactions can be expected. For non-premixed combustion, *τ*_*c*_ represents a measure for the rate at which the chemistry progresses, since the flame propagation speed is limited by mixing. For premixed combustion, however, *τ*_*c*_ relates to the flame structure, which allows us to distinguish between further regimes such as wrinkled and corrugated flamelets, thin and broken reaction zones and well-stirred reactors. The influence of the Kolmogorov scales is characterized by the Ka number, K*a*_*t*_ = *τ*_*c*_/*τ*_*K*_, in which *τ*_*K*_ is the Kolmogorov time-scale. For Ka < 1, *τ*_*c*_ < *τ*_*K*_, which limits the interactions between turbulence and chemistry. The turbulence can thus only moderate the shape of the flame front. Depending on the ratio $v^{\prime }/s_{u}$, wrinkled or corrugated flamelets occur: for small *v*′ the flame may only be wrinkled, whereas for larger *v*′ strong interactions with the flame may occur, which then leads to convoluted and disturbed flame fronts. For this case the mean turbulent Re, Da and Ka-numbers are Re_*t*_ ≈ 2700, Da_*t*_ ≈ 16.5 and Ka_*t*_ ≈ 14.5, positioning this case in the thin reaction zone regime. The range of Re_*t*_, Da_*t*_ and Ka_*t*_ numbers spans three to four orders of magnitudes, indicating that a wide span of physics can be expected. From Fig. 6a we find that the heat-release mainly takes place in the thin reaction zone, but post-flame combustion also occurs at 1 < Da_*t*_ < 5. We also find that regions of high CH_4_ are placed between the post-flame region, rich in CO_2_, and the primary flame region, rich in CO and CO_2_, as well as in radicals. Figure 6b indicates that the main flame is premixed (cf. Fig. 5d), which is also corroborated by the distribution of CH_4_, CO and CO_2_ in the Re_*t*_-Da_*t*_ plane. Mixing occupies a substantial region of the Re_*t*_-Da_*t*_ plane.

**Fig. 7:**
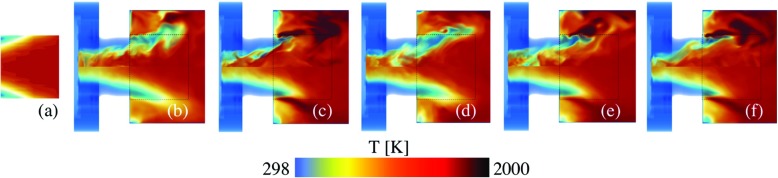
Velocity comparison between **a** experimental PIV data and LES using the **b** EDC, **c** TFM, **d** PaSR, **e** SF and **f** ADM models, using SG35. The *upper half* of the panels shows instantaneous data and the *lower half* of the panels shows time-averaged data. The *dashed box* corresponds to the patch studied experimentally, separately depicted in Fig. 7a

**Fig. 8:**
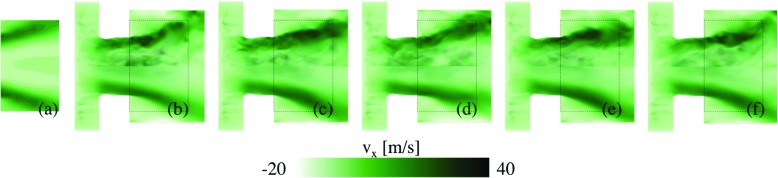
Temperature comparison between **a** experimental Raman scattering data and LES using the **b** EDC, **c** TFM, **d** PaSR, **e** SF and **f** ADM models, using SG35. The *upper half* of the panels shows instantaneous data and the *lower half* of the panels shows time-averaged data. The *dashed box* corresponds to the patch studied experimentally, separately depicted in Fig. 8a

**Fig. 9:**
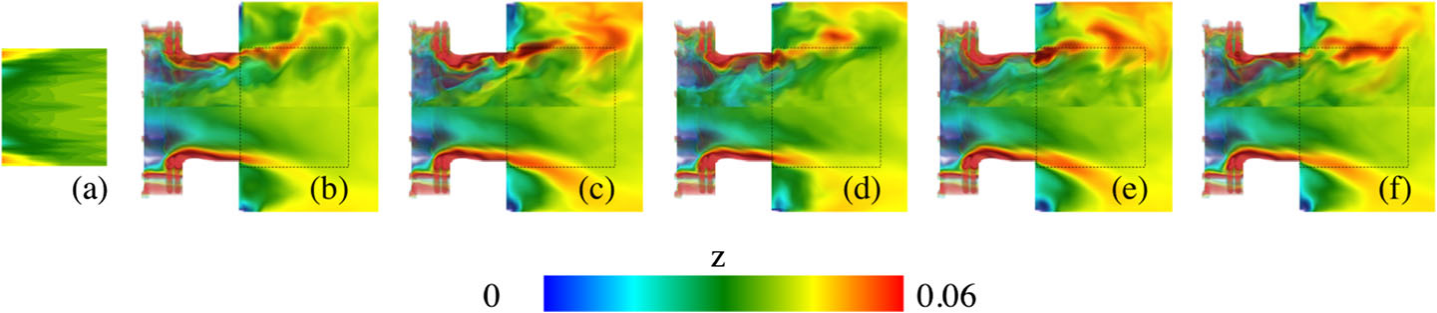
Mixture fraction comparison between **a** experimental Raman scattering data and LES using the **b** EDC, **c** TFM, **d** PaSR, **e** SF and **f** ADM models, using SG35. The *upper half* of the panels shows instantaneous data and the *lower half* of the panels shows time-averaged data. The *dashed box* corresponds to the patch studied experimentally, separately depicted in Fig. 9a

**Fig. 10:**
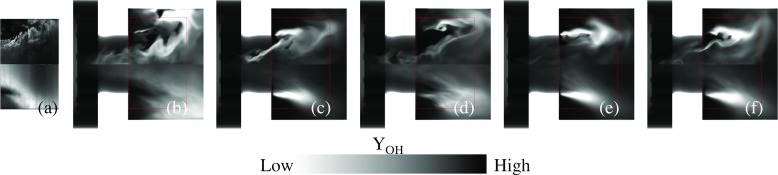
OH concentration comparison between **a** experimental OH-PLIF data and LES using the **b** EDC, **c** TFM, **d** PaSR, **e** SF and **f** ADM models, using SG35. The *upper half* of the panels shows instantaneous data and the *lower half* of the panels shows the time-averaged data. The *dashed box* corresponds to the patch studied experimentally, separately depicted in Fig. 10a

**Fig. 11:**
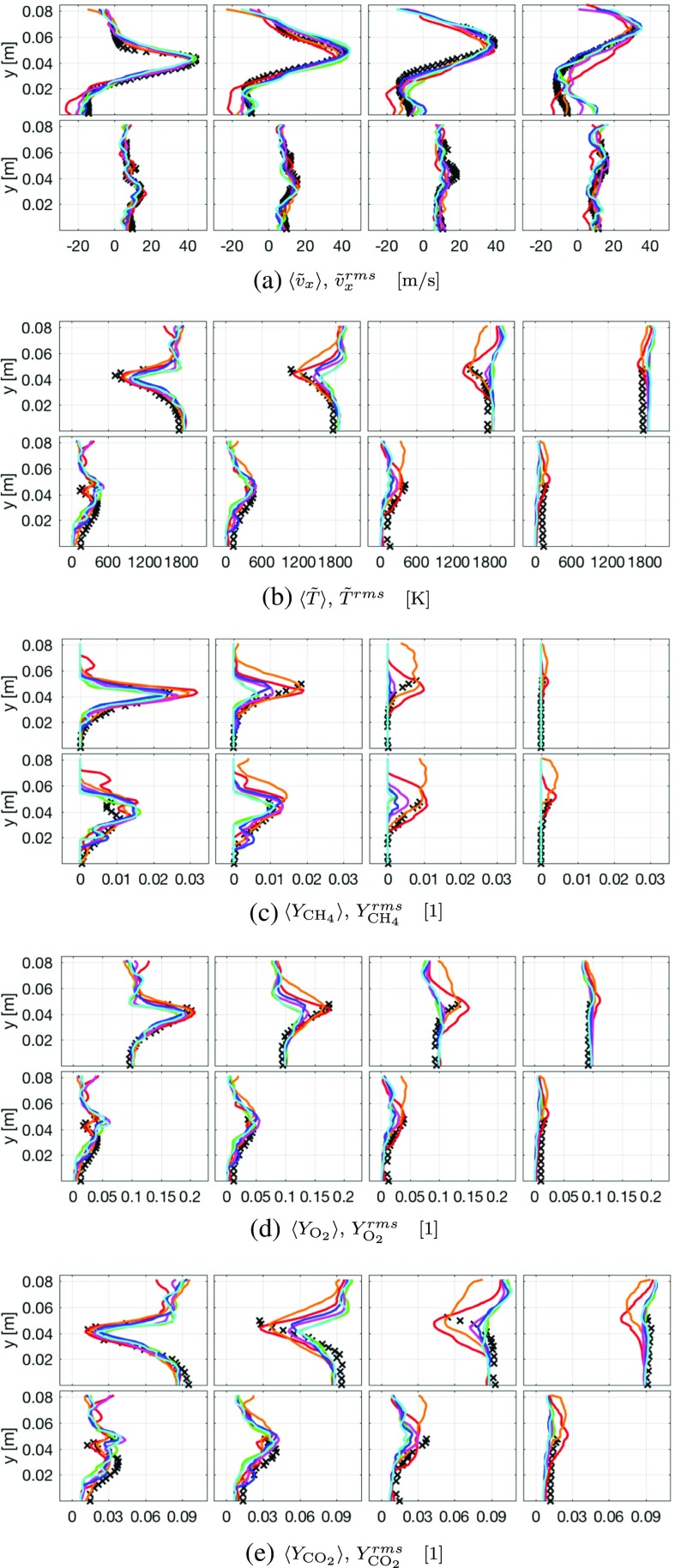
Comparison of measured and predicted mean (*top*) and rms fluctuations (*bottom*) on the 7 Mcell grid. Legend: () LES-PaSR, () LES-EDC, () LES-FM, () LES-TFM, () LES-SF, () LES-ADM and (+ ) experimental data from [49–51]. Only upper half of combustor is shown

Figures 7, 8, 9 and 10 provide a qualitative comparison of the instantaneous (upper half of panels) and time-averaged (lower half of panels) axial velocity, *v*_*x*_, temperature, *T*, mixture fraction, *z*, and OH mass-fractions, *Y*_OH_, from the LES-EDC, TFM, PaSR, FM, SF and ADM models using the SG35 reaction mechanism. The corresponding mean experimental data is shown in the left-most panels of Figs. 7 to 10, respectively. These images are obtained from the experimental data in the four cross-sections x/D = 1.21, 1.44, 1.66 and 2.00, Fig. 1, using cubic spline interpolation, and are mainly intended to provide an overview of how the flow and flame behave just downstream of the burner in the region indicated by the dashed lines in panels (b) to (f). A more detailed quantitative comparison is provided in Fig. 11.

Figure 7 presents the instantaneous (upper half of panels) and time-averaged (lower half of panels) axial velocity components, $\tilde {v}_{x}$ and $\langle \tilde {v}_{x}\rangle $, respectively, from the LES-EDC, TFM, PaSR, FM (not shown), SF, and ADM models. The justification for not presenting the LES-FM result is that these are very similar the LES-PaSR results. The predicted velocities largely agree well with each other, and with the experimental mean axial velocity, 〈*v*_*x*_〉. Both $\tilde {v}_{x}$ and $\langle \tilde {v}_{x}\rangle $ show a high-speed, annular, funnel-shaped, axial velocity region discharging from the burner, enclosing the inner recirculation region, resulting from the axisymmetric vortex breakdown, and limiting the outer recirculation region established between the discharging fuel-air mixture and the combustor dump plane and side wall. Significant unsteadiness in the velocity is observed by comparing $\langle \tilde {v}_{x}\rangle $ and $\tilde {v}_{x}$ as a consequence of the unsteady, swirling, inner and outer shear-layers restricting the high-velocity regions. The differences between the LES model predictions are surprisingly small, primarily constrained to somewhat different shear-layer thickness growth and peak $\langle \tilde {v}_{x}\rangle $. Compared to the experimental data, good agreement is generally observed.

Figure 8 shows instantaneous (upper half of panels) and time-averaged (lower half of panels) temperatures, $\tilde {T}$ and $\langle \tilde {T}\rangle $, respectively, from the models presented in Fig. 7. The predicted temperatures agree well with each other, and with the experimental mean temperature, 〈*T*〉, but showing larger model-to-model variations compared to $\tilde {v}_{x}$ and $\langle \tilde {v}_{x}\rangle $. Both $\tilde {T}$ and $\langle \tilde {T}\rangle $ reveal the M-shaped flame structure discussed in Figs. 4 and 5, and the significance of the LES combustion model. The differences between the LES models are primarily limited to the size and shape of the fuel-air cloud discharging into the combustor. As the reaction zones are largely located in the inner and outer shear layers, Fig. 5, and the annular tip at which these meet, the observed differences can be attributed to how the filtered reaction rates are modeled, and how these terms interact with the molecular transport terms. Based on the 〈*T*〉 distribution, the LES results can be divided into two groups: one group consists of the LES-EDC, PaSR and FM models, resulting in a slightly larger M-shaped flame, and one group consists of the LES-TFM, SF and ADM models, resulting in a slightly smaller M-shaped flame. Compared to the experimental data, good agreement is generally observed for both groups of models with only small differences, mainly related to the size and detailed shape of the time-averaged flame.

Figure 9 shows instantaneous (upper half of panels) and time-averaged (lower half of panels) mixture fractions, $\tilde {z}$ and $\langle \tilde {z}\rangle $, respectively, from the models listed above. The mixture fraction *z* = (*β* − *β*_*ox*_)/(*β*_*fu*_ − *β*_*ox*_) measures the degree of mixing, in which *β*_*fu*_ and *β*_*ox*_ are coefficients in the fuel and oxidizer streams, respectively, and the coupling function *β* is defined by the elemental mass fractions, $z_{\alpha } = {\sum }_{k=1}^{N_{s}}\alpha _{\alpha ,k}(M_{\alpha }/M_{k})Y_{k}$, in which *N*_*s*_ is the number of species, *α*_*α*,*k*_ the number of atoms of element *α* in specie *k*, *M*_*α*_ and *M*_*k*_ the molecular weight of element *α* and specie *k*, such that $\beta ={\sum }_{\alpha =1}^{N_{e}}\gamma _{\alpha } z_{\alpha }$, where *γ*_*α*_ are weighting factors. The *γ*_*α*_s are not unique, and several different values are used, [83]. Here, we adopt the definition of Stopper et al. [51], *γ*_C_ = 2/*M*_C_, *γ*_O_ = −1/*M*_O_ and *γ*_H_ = 1/2*M*_H_, so that Bilger’s original definition of the mixture fraction, *z*, [88], is recovered. The predicted mixture fractions agree well with each other, and with the average experimental mixture fraction, 〈*z*〉. The highest values of $\tilde {z}$ and $\langle \tilde {z}\rangle $ are found in the fuel rich streams from the fuel inlets that are located adjacent to the pre-chamber walls due to the imposed swirl. Significant inhomogeneity in $\tilde {z}$ is observed, whereas $\langle \tilde {z}\rangle $ results in gradually decreasing values along the swirling path of the discharging fuel-air mixture. Low values of $\langle \tilde {z}\rangle $ and $\tilde {z}$ are observed in the inner recirculation region, extending into the pre-chamber. Similar distributions of $\langle \tilde {z}\rangle $ are obtained from all LES models, differing only slightly from the experimental $\langle \tilde {z}\rangle $ distribution in terms of the extent of the peak values of the mixture fraction.

Figure 10 shows instantaneous (upper half of panels) and time-averaged (lower half of panels) OH concentrations, $\tilde {Y}_{\text {OH}}$ and $\langle \tilde {Y}_{\text {OH}}\rangle $, respectively, from the models presented. The predicted OH concentrations show larger model-to-model differences than any other quantities. In addition, the differences between the model predictions and the experimental data are larger for OH than for any other quantity examined. In a laminar (premixed) flame OH typically starts to form at the temperature inflexion point in the inner layer, then rapidly increases to peak in the oxidation layer, after which it slowly decays into the hot CO, CO_2_ and H_2_O rich region of the flame. Here, OH exists in the inner recirculation region, in the pre-chamber flow, and outside of the swirling fuel-air mixture discharging into the combustor. The instantaneous results reveal significant unsteadiness, turbulence chemistry interactions, local extinction, and strain effects due to intense vortical shear-layer structures interacting with the flame. The time-averaged results present differences compared with the experimental OH-PLIF images that are deemed related to the filtered reaction rate modeling and its interactions with the molecular transport. The LES predictions can be divided into two groups: one group consisting of the LES-EDC, PaSR and FM models, which show burning in the inner shear-layer and intermittent burning in the outer shear-layer, resulting in a detached OH profile that is in agreement with the OH-PLIF data. The second group consists of the LES-TFM, SF and ADM models, which also display burning in the inner shear-layer but a more constant and intense burning in the outer shear layer.

**Fig. 12:**
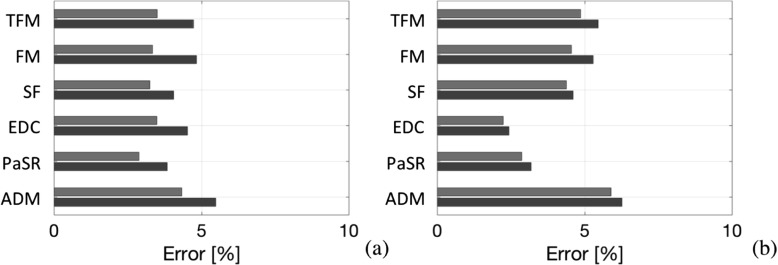
Error bar charts of the **a** axial velocity, *v*_*x*_, and **b** temperature, *T*, showing the difference between LES combustion predictions on the baseline (*dark gray*) and fine (*light gray*) grids for all LES combustion models examined in this study

Figure 11 provides a quantitative comparison of the time-averaged and rms fluctuations of the axial velocity, $\tilde {v}_{x}$; temperature, $\tilde {T}$; methane (CH_4_) mass fraction, $\tilde {Y}_{\mathrm {CH_{4}}}$; oxygen (O_2_) mass fraction, $\tilde {Y}_{\mathrm {O2}}$; and carbon dioxide (CO_2_) mass fraction, $\tilde {Y}_{\mathrm {CO_{2}}}$; from the experimental data and from the LES-EDC, TFM, PaSR, FM, SF and ADM models at the cross-sections x/D = 1.21, 1.44, 1.66 and 2.00. Due to the shape of the combustor and the length of the statistical sampling the mean and rms-fluctuation profiles are symmetric over the combustor centerline. The LES-PaSR model was used to evolve the flow from its initial conditions for ∼10 flow-through times (∼60 ms) until the flow has reached its fully developed nature, after which another ∼5 flow-through times was used to further develop the flow with the different LES combustion models, after which statistical averaging was carried out for another ∼10 flow-through times. Only results from the baseline grid are presented in Fig. 11, whereas the influence of the grid resolution is discussed in more detail in Fig. 12. Figure 11 reveals that the LES predictions generally agree favourably with the measured data, as however already evident in Figs. 7 to 10, but with some key differences particular to the individual LES combustion models outlined in Section 3.

Regarding the mean axial velocity, $\langle \tilde {v}_{x}\rangle $, and its axial rms fluctuations, $\tilde {v}_{x}^{rms}$, in Fig. 11a we find that the velocity initially peaks at around y ≈±0.043 m, which corresponds to the location of the upper/lower burner exit, and gradually expands radially at the same time as the peak value decreases and the profile widens. The velocity profiles also reveal the central recirculation region ending around or just downstream of x/D = 2.00. No explicit evidence is found in $\langle \tilde {v}_{x}\rangle $ regarding the outer recirculation region observed in $\tilde {v}_{x}$ for all LES model predictions, hence corroborating that this structure is intermittent, and thus almost non-existent in $\langle \tilde {v}_{x}\rangle $. The flow is highly turbulent as is indicated by high axial rms fluctuations, $\tilde {v}_{x}^{rms}$, with values approaching 25% of the peak mean axial velocity, $\langle \tilde {v}_{x}\rangle $. The selection of LES combustion models does not influence the overall structure of $\langle \tilde {v}_{x}\rangle $ and $\tilde {v}_{x}^{rms}$, but rather details thereof: (i) the LES-PaSR model appears to overpredict the strength of the recirculation region and tends to predict a somewhat too narrow velocity funnel, (ii) the LES-TFM, SF and ADM models, and to some extent also the LES-FM model, tend to predict a somewhat too short central recirculation region, with flow acceleration starting around x/D = 2.00. (iii) The LES-EDC model seems to be the model that exhibits best agreement with the experimental measurement data, although slightly underestimating the peak values of 〈*v*_*x*_〉, and resulting in a slightly too narrow velocity funnel. The rms-velocity fluctuations are predicted reasonably by all LES combustion models, with the exception of the LES-EDC model predicting somewhat higher fluctuation levels than the other models examined.

The mean temperature, $\langle \tilde {T}\rangle $, and temperature rms fluctuations, $\tilde {T}^{rms}$, in Fig. 11b also generally show good agreement with the experimental data, but demonstrate a larger spread between LES-model predictions. The mean combustor temperature, determined by the global stoichiometry, is well predicted by all LES combustion models, being about 20 K in excess of the measured temperature (at x/D = 2.00) possibly due to lack of thermal radiation models that can adequately handle the influence of the quartz windows. The cold fuel-air mixture discharging into the combustor by the high-speed (M-shaped) velocity funnel results in an M-shaped region of lower $\langle \tilde {T}\rangle $ in the beginning of the combustor that, however, gradually approaches the mean combustor temperature, so that at x/D=2.00 $\langle \tilde {T}\rangle $ is almost constant across the combustor. The different LES combustion models result in different $\langle \tilde {T}\rangle $ recovery rates, with the LES-TFM, SF and ADM models showing a faster return to the mean combustor temperature than the LES-PaSR and EDC models that also agree better with the experimental temperature profiles. The LES-EDC model, however, reveals a wider flame outside of the velocity funnel. The $\langle \tilde {T}\rangle $ profiles of the LES-SF model agrees well with those presented by Bulat et al. [44], suggesting that at least this model behaves similarly irrespective of subgrid flow models and numerical methods. Regarding $\tilde {T}^{rms}$ it is noted that the predictions from the LES-PaSR and EDC models show slightly better agreement with the measurement data than the other models, particularly at x/D = 1.21.

Good agreement between measured data and LES results are also found for $\langle \tilde {Y}_{\text {CH}_{4}}\rangle $, $\langle \tilde {Y}_{\mathrm {O}_{2}}\rangle $ and $\langle \tilde {Y}_{\text {CO}_{2}}\rangle $ and their rms-fluctuations $\tilde {Y}_{\text {CH}_{4}}^{rms}$, $\tilde {Y}_{\mathrm {O}_{2}}^{rms}$ and $\tilde {Y}_{\text {CO}_{2}}^{rms}$ in Fig. 11c, d and e, respectively. The average reactant mass-fractions $\langle \tilde {Y}_{\text {CH}_{4}}\rangle $ and $\langle \tilde {Y}_{\mathrm {O}_{2}}\rangle $, as well as their rms-fluctuations, $\tilde {Y}_{\text {CH}_{4}}^{rms}$ and $\tilde {Y}_{\mathrm {O}_{2}}^{rms}$, behave similarly, being high in the high-speed (M-shaped) velocity funnel consisting of reactants discharging into the combustor. The predictions from the LES-TFM, FM, SF and ADM models show good agreement with the measurement data at x/D = 1.21 and 2.00, but are consumed too fast as apparent by the two middle panels (x/D = 1.44 and 1.66) of Fig. 11c and d. The LES-PaSR and EDC models overpredict $\langle Y_{\text {CH}_{4}}\rangle $ slightly at x/D = 1.21 but predict the consumption of $\langle Y_{\text {CH}_{4}}\rangle $ and $\langle Y_{\mathrm {O}_{2}}\rangle $ slightly better than the other models. The LES-EDC model, however, predicts wider $\langle \tilde {Y}_{\text {CH}_{4}}\rangle $ and $\langle \tilde {Y}_{\mathrm {O}_{2}}\rangle $-profiles at x/D = 1.66, but since no experimental data are available in this regime this behavior cannot be quantified. The rms-fluctuations, $\tilde {Y}_{\text {CH}_{4}}^{rms}$ and $\tilde {Y}_{\mathrm {O}_{2}}^{rms}$ show acceptable agreement with the measurement data, with the LES-PaSR and EDC models showing better agreement in the details. Regarding $\langle \tilde {Y}_{\text {CO}_{2}}\rangle $ and $\tilde {Y}_{\text {CO}_{2}}^{rms}$ in Fig. 11e good agreement for all models are observed at x/D = 1.21 and 2.00, whereas some deviations are found at x/D = 1.44 and 1.66. Here, the LES-PaSR and EDC models show better agreement with the experimental data than the LES-TFM, FM, SF and ADM models, the dissimilarity being the difference in production rate of CO_2_, and conversely the consumption rates of CH_4_ and O_2_. All in all we find that the LES combustion models perform reasonably, although with room for improvements, particularly with respect to the filtered reaction rate modelling.

To represent the prediction quality achieved in this study we follow the approach suggested by Ma et al. [47, 48], and introduce an error estimate, $E=\frac {1}{M}{\sum }_{k=1}^{M}\left (\sqrt {\frac {1}{N_{k}}{\sum }_{i=1}^{N_{k}}\left (\langle \tilde {\phi }_{i,k}\rangle -\langle \phi _{i,k}\rangle \right )^{2}}\right )$, where $\langle \tilde {\phi }_{i,k}\rangle $ denotes the time-averaged values of the variable *ϕ* from the LES, 〈*ϕ*_*i*,*k*_〉 the mean values from the experiments, *N*_*k*_ the number of experimental measurement points, *i*, per cross-section, *k*, and *M* the number of cross-sections. Error estimates are calculated for the axial velocity, *v*_*x*_, and the temperature, *T*, for all models as illustrated in Fig. 12a and b, respectively. In Fig. 12 these errors estimates are normalized with the time-averaged axial velocity and temperature. Most models perform within a relatively narrow error range of less than 9% for *v*_*x*_, and 6% for *T*, which is found to decrease with approximately 2% on the finer grid. The experimental investigations report both precision and accuracy errors, resulting in a total error of about 5% for *v*_*x*_, and 10% for *T*, [49–51], making it virtually impossible to select a ’best-performing’ LES combustion model, as all of the models compared essentially perform within the experimental uncertainty regime. Regarding the axial velocity, *v*_*x*_, in Fig. 12a we find that all models perform equally well showing error estimates of ∼5% that reduces to ∼4% on the fine grid. Regarding the temperature, *T*, in Fig. 12b we find a larger spread between the models, with the LES-ADM model showing the largest error estimate of about 6% and the LES-PaSR and EDC models showing the smallest error estimate of about 3%, whereas the LES-TFM, FM and SF models perform similarly, with an error between 4% and 5%. The influence of grid resolution is also less apparent for *T* than for *v*_*x*_. As evident from Fig. 11 the spread across cross-sections is significant, and this comparison only provides an overall estimate of the error in the measured part of the flame. Within this range of models, there is no strong case for deciding which model performs the best. However, it is more apparent in Fig. 12 than in Fig. 11 that there is a significant influence of the LES combustion model on the axial velocity, *v*_*x*_, that is not easily understood.

## Concluding Remarks

Here, we report on an investigation of a lean premixed natural gas-air swirl flame in an industrial gas turbine combustor, [49–51], that has been performed aiming at comparing different finite rate chemistry LES combustion models based on the 35-step skeletal Smooke and Giovangigli reaction mechanism, [76]. The finite rate chemistry LES combustion models studied include the Thickened Flame Model (TFM), [55], the Eddy Dissipation Concept (EDC) approach, [39], the Partially Stirred Reactor (PaSR) model, [41], the Fractal Model (FM), [40], the Approximate Deconvolution Model (ADM), [42], and the Stochastic Fields (SF) model, [44, 64]. These models are all implemented in an in-house developed solver based on OpenFOAM, [67], and the equations are solved using a high-order monotonicity preserving convective recostruction algorithm, central differencing and Crank-Nicholson time-integration, [68], combined with a Strang-type, Rosenbrock, operator-splitting scheme, [69], for integrating the combustion chemistry. Regarding the computational cost we find the cost of all models, with exception of the SF model, very similar. The cost of the SF model increases with increasing number of stochastic fields used, and for the number of stochastic fields used here, this model is about 60% more expensive than the other models.

In the paper reported, we take advantage of the multiple combustion predictions available and describe the flow and flame dynamics in detail before we qualitatively and quantitatively examine the influence of the finite rate chemistry LES combustion models. Detailed comparisons are made with velocity, temperature and available species experimental data. The overall flow and flame structures appear similar for all LES combustion models studied and agree well with experimental still and video images. The flame takes the shape of an M-shaped structure that wraps around the premixed fuel-air cloud as it rotates around the combustor axis due to the imposed swirl whilst being wrinkled by the turbulence. The heat-release originates from multiple thin layers of interpenetrating reaction structures and layers of species defined by the reaction mechanism. The flame is composed of an inner, wrinkled, V-shaped flame located in the inner shear-layer of the discharging fuel-air mixture, and an outer swirling flame, taking the shape of a truncated wrenched cone located in the outer shear-layer of the discharging fuel-air mixture. The two flame elements connect to each other at the swirling annular flame tip, located between 0.5D and 0.8D downstream of the burner exit depending on the LES combustion model, but also on the flame dynamics. Takeno flame index analysis and chemical explosives mode analysis suggests that the flame is premixed and resides within the thin reaction zone in the Williams diagram of combustion. The LES results show good agreement with the experimental data for the axial velocity, temperature and major species, but differences due to the selection of LES combustion model are observed and discussed. All models studied perform within the experimental uncertainty regime although somewhat differently: the LES-EDC, PaSR and FM models typically result in a slightly larger flame whereas the LES-TFM, SF and ADM models typically result in a slightly smaller flame. Within the range of models examined, there is however no strong case for deciding which model performs the best. Additional cases should be studied to give a broader perspective of the case sensitivity of the models. In spite of this, there is still room for development of new, more accurate and efficient, combustion models.
